# Leishmanicidal Activity of Biogenic Fe_3_O_4_ Nanoparticles

**DOI:** 10.3390/scipharm85040036

**Published:** 2017-11-20

**Authors:** Mehrdad Khatami, Hajar Alijani, Iraj Sharifi, Fatemeh Sharifi, Shahram Pourseyedi, Sam Kharazi, Marcos Augusto Lima Nobre, Manouchehr Khatami

**Affiliations:** 1 School of Medicine, Bam University of Medical Sciences, Bam, Iran; Mehrdad7Khatami@gmail.com (M.K.); Kharazi@gmail.com (S.K.); 2 Nanomedicine and Nanobiology Research Center, Shiraz University of Medical Sciences, Shiraz, Iran; 3 Leishmaniasis Research Center, Kerman University of Medical Sciences, Kerman, Iran; 4 Pharmaceutics Research Center, Institute of Neuropharmacology, Kerman University of Medical Sciences, Kerman, Iran; fatemeh7267@gmail.com; 5 Department of Biotechnology, Shahid Bahonar University of Kerman, Kerman, Iran; spseyedi@gmail.com; 6 Fac de Ciências e Tecnologia-FCT, Universidade Estadual Paulista-UNESP, Presidente Prudente-SP 19060-900, Brazil; nobremal@fct.unesp.br; 7 Department of Radiology, Kerman University of Medical Sciences, Kerman, Iran; M.Khatami@kum.ac.ir

**Keywords:** biosynthesis, iron oxide nanoparticles, IONPs, leishmanicidal

## Abstract

Due to the multiplicity of useful applications of metal oxide nanoparticles (ONPs) in medicine are growing exponentially, in this study, Fe_3_O_4_ (iron oxide) nanoparticles (IONPs) were biosynthesized using Rosemary to evaluate the leishmanicidal efficiency of green synthesized IONPs. This is the first report of the leishmanicidal efficiency of green synthesized IONPs against *Leishmania major.* The resulting biosynthesized IONPs were characterized by ultraviolet-visible spectroscopy (UV-Vis), X-ray diffraction (XRD), transmission electron microscopy (TEM), and Fourier transform infrared spectroscopy (FTIR). The leishmanicidal activity of IONPS was studied via 3-4,5-dimethylthiazol-2-yl)-2,5-diphenyltetrazolium bromide (MTT) assay. The results showed the fabrication of the spherical shape of monodisperse IONPs with a size 4 ± 2 nm. The UV-visible spectrophotometer absorption peak was at 334 nm. The leishmanicidal activity of biogenic iron oxide nanoparticles against *Leishmania major* (promastigote) was also studied. The IC_50_ of IONPs was 350 µg/mL. In this report, IONPs were synthesized via a green method. IONPs are mainly spherical and homogeneous, with an average size of about 4 nm, and were synthesized here using an eco-friendly, simple, and inexpensive method.

## 1. Introduction

Misuse of antibiotics may lead to the spread of resistant microbial strains. The increase in resistant microbial strains is becoming a serious and complex problem in medicine; therefore, it is necessary to explore new antimicrobial substances and replace them with common antibiotics [1]. Nanoparticles have attracted scientists’ interests due to their strong antimicrobial properties [2,3]. Banach et al. [4] successfully studied the bactericidal and fungicidal effect of metallic nanoparticle in real conditions.

Nanobiotechnology is an emerging field of nanotechnology that utilizes nano-bio-based systems for such biological usages as the therapeutic drug delivery, immunology, biotechnology, medicine, and engineering [5,6,7,8,9]. According to the European Commission recommendations, nanoparticles are defined as manufactured materials containing particles in a free state or as an aggregate, wherein at least 50% of the particles have one or more dimensions in the range of 1–100 nm [10,11]. Nowadays, nanoparticles are the most important novel drag or gene vectors for cells that can contribute to the development of drug delivery, medicine, and genetics [12,13,14,15]. Nanostructures are categorized into different types, such as nanotubes, nanoparticles, nanorods, nanowires, and nanosheets, based on size and shape [16,17,18].

Due to the unique physicochemical, thermal, and optical properties and good surface characteristics, magnetic nanoparticles are used in many branches of biomedical sciences, such as magnetic resonance imaging (MRI) [19] and brain cancer therapy [20]. For example, magnetic nanoparticles can accumulate in tumor tissue and play an important role in the detection and treatment of cancer through electron microscopes, MRI imaging, or drug delivery [21,22].

One reason for the attraction of magnetic nanoparticles is their potential application in medicine, health, and environment such as the development of enzyme performance [23,24], the absorption and deletion of heavy metals [23,24], and dye [25] from contaminated wastewater. Most research has been conducted on magnetic nanoparticles of iron, cobalt, and nickel [26]. The synthesis of metal oxide nanoparticles (ONPs) has been carried out via various physical and chemical methods [27,28,29]. The chemical methods possess potential environmental and human health risks. Synthesis of magnetic nanoparticles using biological resources is called biosynthesis or green synthesis, which is environmentally friendly and a safe method for the synthesis of biological and natural compounds such as intracellular/extracellular extracts including non-harmful microbial, plant, or fungi compounds [30]. Magnetic nanoparticles of iron have low toxicity, are biocompatible, and are considered paramagnetic nanoparticles that are widely used in many branches of medical sciences, such as radiology [31,32], biosensors [31], and drug delivery [32]. The tremendous advantage of metal oxide nanoparticles is their isolatory nature and their easy collectability using an external magnetic field, which makes the recovery and re-use as simple as possible [23,33].

The current study was conducted to synthesize leishmanicidal iron oxide nanoparticles (IONPs) using *Rosmarinus officinalis*, which is a renewable biological resource. *R. officinalis* has high antioxidants [34], and it is used as a safe food flavoring due to its favorable taste and aroma [35].

Therefore, in this study, we aimed to evaluate the leishmanicidal efficiency of biosynthesized IONPs.

## 2. Material and Methods

### 2.1. Biosynthesis of Fe_3_O_4_ Nanoparticles

*R. officinalis* leaves were obtained from the local market of Kerman city. Ten grams of leaves were washed with sterile double distilled deionized water (SDDW), dried at air temperature (28 °C), and powdered with mortar; then, the powder was added to the Erlenmeyer containing 1000 mL of SDDW. The final mixture was heated (70–80 °C) for 30 min [36]. The mixture was centrifuged at 1118 rcf for 5 min, and the supernatant was collected for further processing. All steps were carried out inside the laminar air-flow cabinet. Therefore, a sterile condition was maintained during the experiments.

The ferric (III) chloride hexahydrate (FeCl_3_·6H_2_O, 98%) stock solution (0.1 M) was prepared by adding 1000 mL of SDDW to 27.03 g of FeCl_3_·6H_2_O, 98%. For the synthesis of iron oxide (Fe_3_O_4_) nanoparticles (NPs), 10 mL of filtered *R. officinalis* extract (FROE) was mixed to a 1 mM FeCl_3_ solution under constant stirring at room temperature. Within a particular time, a change in color from light yellow to black was achieved via synthesis of IONPs [37].

### 2.2. Characterization Methods and Instruments

The UV-visible spectra were recorded over the 300–650 nm range with a Scan Drop-type product, (Analytik jena, Berlin, Germany). Transmission electron microscopy (TEM) observations were carried out on a Carl ZEISS EM10C, 80 kV. Fourier transform infrared spectroscopy (FTIR) spectra (Tensor 27, Analytik jena, Berlin, Germany) of the IONPs were recorded over a range of 500–3500 cm^−1^ on a model spectrum of 100 series spectrophotometer. The crystalline structure and phase purity of the IONPs produced were identified via X-ray diffraction measurement (STOE Stidy-mp, (λ = 1.541 Å) in the region of 2θ from 10° to 80° [38].

Magnetic properties of the samples were measured using vibrating sample magnetometry (VSM), Lake Shore Model 7400 nder magnetic fields up to 10 kOe.

The effect of IONPs on *Leishmania major* promastigotes was evaluated with a colorimetric cell viability 3-4,5-dimethylthiazol-2-yl)-2,5-diphenyltetrazolium bromide (MTT) assay using the method described by Pro. Mahmoudvand et al. [39]. Briefly, 100 µL of the promastigotes (10^6^ cells/mL) harvested from the logarithmic growth phase was added to a 96-well microtiter plate. One hundred microliters of various concentrations (0–400 µg/mL) of IONPs were then added to each well and incubated at 25 °C for 72 h. After incubation, 10 µL of MTT solution (5 mg/mL) was added to each well and incubated at 25 °C for 4 h. Promastigotes were cultured in a complete medium with no IONPs, used as an untreated control, a complete medium with no promastigotes or IONPs, used as a blank. All experiments were performed three times. Finally, absorbance was measured by an ELISA reader (BioTekELX800, Winooski, VT, USA) at 490 nm. Fifty percent inhibitory concentrations (IC_50_ values) were also calculated via a Probit test in SPSS (IBM SPSS Statistics V22.0, Chicago, IL, USA).

Data analysis was carried out using SPSS statistical package version 17.0. Differences between the test and control groups were analyzed via *t*-test and ANOVA. *p <* 0.05 was considered as statistically significant. IC_50_ (50% inhibitory concentrations) were calculated via a Probit test in SPSS.

## 3. Results

### 3.1. Characterization

**Figure 1:**
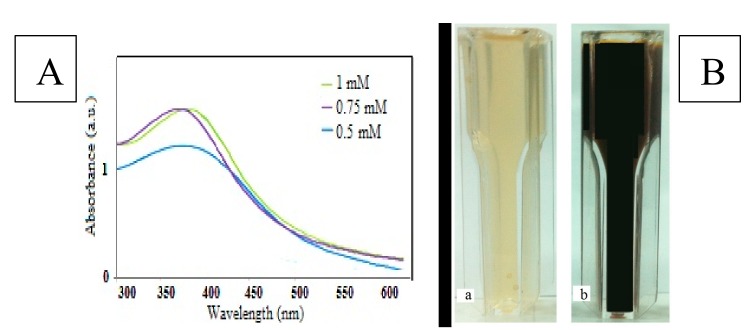
(**A**) UV-visible absorption spectra of *R. officinalis* extract. (**B**) Photograph of samples change color. a: *Rosmarinus officinalis* extract; b: iron oxide (Fe_3_O_4_) nanoparticles (IONPs).

The UV-Vis spectrum, shown in Figure 1A, revealed an absorption peak between 334 and 367 nm. The color of the FROE changed to black (Figure 1B) at standard conditions. Discoloration was the first visible sign of biosynthesis of IONPs.

The effect of different concentrations of iron ion precursors, extract, and time (at constant temperature and pH) represents the functionality of the FROE in response to the synthesis process. Increase in all three parameters led to an increase in the intensity peak of UV-visible analysis that shows the better effect of increasing these parameters on the synthesis process. The primary effect of different concentrations of metal ions on synthesizing nanoparticles showed that the best absorption peak is a sample treated with a 1 mM concentration of iron chloride.

**Figure 2:**
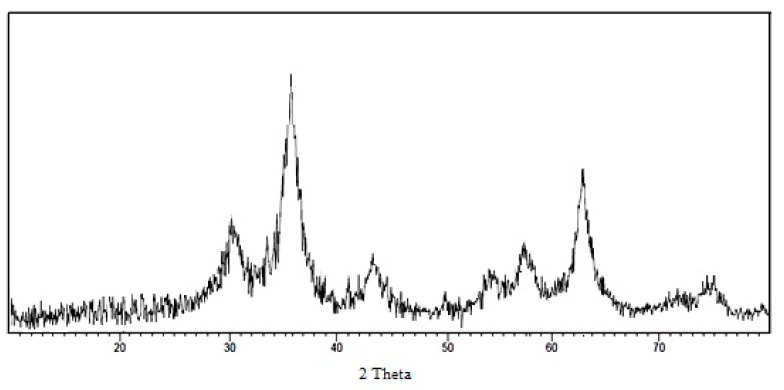
X-ray diffraction pattern of biosynthesized IONPs using *Rosmarinus officinalis.*

The X-ray diffraction (XRD) spectrum of the synthesized IONPs by FROE is shown in Figure 2. It was found that there were strong diffraction peaks with 2θ values of 30°, 35.8°, 43.3°, 53.9°, 57.4°, 62.9°, and 74.6°, corresponding to the crystal planes of (220), (311), (440), (422), (511), and (440) of the crystalline structure of Fe_3_O_4_ NPs, respectively.

**Figure 3:**
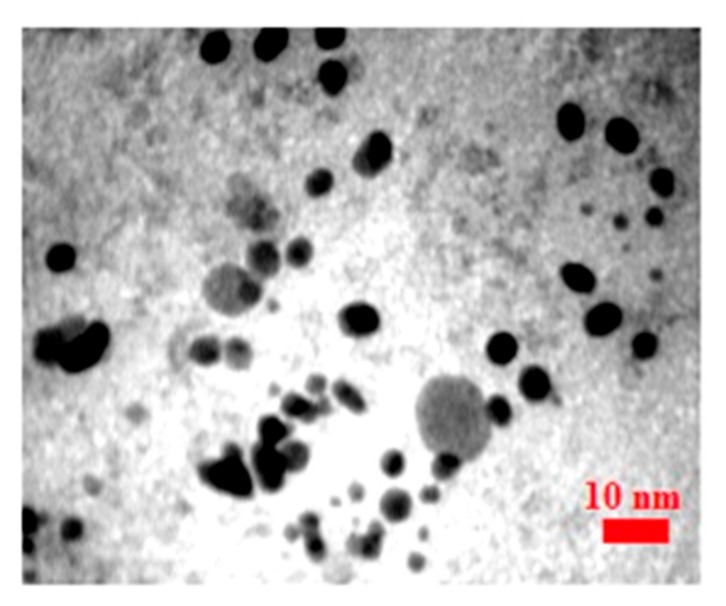
Transmission electron microscopy images and histogram of biosynthesized IONPs.

Figure 3 shows the TEM images and histogram of biosynthesized Fe_3_O_4_ NPs, the particle size range was from 1 to 12 nm. The IONPs were mainly spherical and approximately monodisperse, with an average size 5 nm.

### 3.2. Fourier Transform Infrared Spectroscopy Analysis

**Figure 4:**
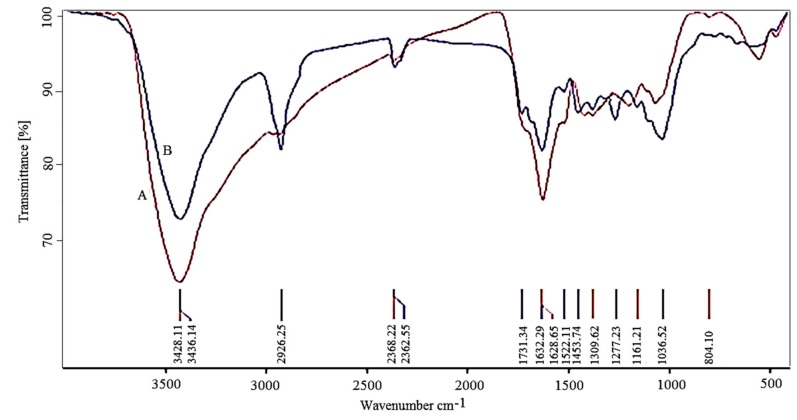
Fourier transform infrared spectroscopy (FTIR) spectrum for the *Rosmarinus officinalis* extracts before (A) and after (B) biosynthesis of iron oxide nanoparticles.

Fourier transform infrared analysis of active functional groups was conducted (Figure 4). The FROE was analyzed before and after the synthesis of IONPs. The possible involvement of active functional groups in the biosynthesis process of IONPs is shown in Table 1.

The band movement from 1632 to 1628 and from 3426 to 3428 cm^−1^ indicate the possible involvement of C–O and C=O groups in the synthesis process. The results showed that water-soluble functional groups of the extract play an important role in the stability of nanoparticles in aqueous suspension.

### 3.3. Antipromastigote Assay

**Figure 5:**
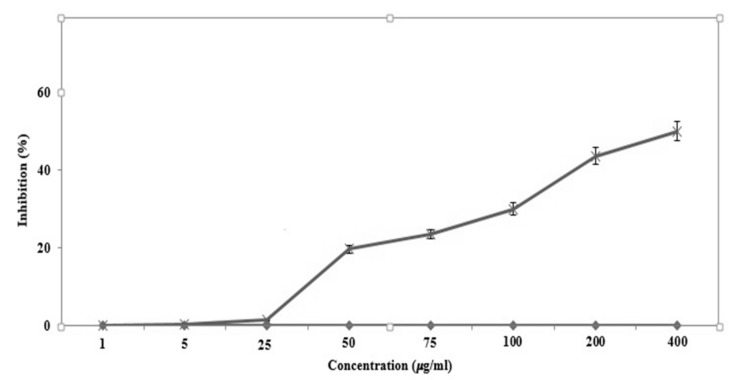
The parasitological effects of IONPs on *Leishmania major* promastigotes.

Leishmaniasis is a significant vector-borne parasitic disease worldwide, caused by the genus *Leishmania*. The objective of the current study was to evaluate the leishmanicidal efficiency of green-synthesized IONPs against *L. major*, one of the species causing cutaneous leishmaniasis, isolated from Iranian patients [46]. The results indicated that the IONPs had antileishmanial activity against the promastigotes of *L. major* based on a dose-dependent response (*p* < 0.001) and the IONPs i showed some antileishmanial effects against promastigotes of *L. major* (Figure 5). The IC_50_ values for the IONPs against promastigotes of *L. major* was 350 µg/mL.

## 4. Discussion

This is the first study of its kind on the leishmanicidal activity of IONPs. This finding is consistent with numerous previous results reported by other investigators who demonstrated that leishmania species promastigotes are significantly less sensitive to pentavalent antileishmanial agents than the respective amastigotes. The findings show that biogenic IONPs has antileishmanial activity against *L. major* (promastigote stage) in a dose-dependent manner (*p* < 0.001). In this study, we green-synthesized IONPs using rosemary, and the primary physic-chemical screening of the synthesized IOPNs showed that the UV-visible absorption peak of IOPNs was about 354. The antileishmania activity of the biosynthesized IOPNs was then studied.

One of the main advantages of this method is producing iron oxide nanoparticles with an average size of about 4 nm; they have a smaller average size compared with the published studies. Sundaram et al. [47] reported the green synthesis of IONPs with an absorption wavelength range between 240 and 350 nm. Additionally, Behera et al. [48] revealed that the UV-visible absorption peak of IOPNs with an average size of 66 was 370 nm. In another study, Arakha et al. [40] found a UV absorption peak of chemically synthesized chitosan-coated IONPs (8 to 20) at 367 nm.

The X-ray diffraction patterns showed strong diffraction peaks at 30°, 35.8°, 43.3°, 53.9°, 57.4°, 62.9°, and 74.6°; this is similar to the results of Parvadan et al. [49] and Arakha et al. [40].

Mahdavi et al. [50] synthesized IONPs with an average size of approximately 18 ± 4 nm. They studied the green synthesis of IONPs by *Sargassum muticum*. Their results were very close to our findings. Additionally, Avad et al. reported the same result with a size of 8–5 nm. However, in UV-visible spectra, the absorption peak was reported at 233 nm, which is different from the results of this study (334 nm). The difference in wavelength is likely due the difference in the size or coated compounds of synthesized IONPs.

Pardan et al. [49] studied the antibacterial effect of IONPs against *E. faecalis*, *P. aeruginosa*, and *E. coli* ATCC 25922. An MIC of about 0.01–0.039 µg/mL was reported. In addition, Tran et al. [51] pointed out that that the growth inhibitory concentration of iron oxide nanoparticles was 3 µg/mL. Another advantage of this method compared to the conventional technique is a reduced production time. In the biosynthesis method (10 s in this study), and in comparison, with physic-chemical methods, less time is needed for the production.

These results were obtained at the laboratory level, but it should be mentioned that the high antileishmania effects of the studied IONPs have no selective effect, and IONPs can be generally effective on all kinds of human cells. Therefore, further studies are required to make these findings applicable and to adjust the iron nanoparticles such that they have special effects on cancer cells. Therefore, we strongly suggest further experimental works to evaluate the effect of IONPs on intra-cellular amastigotes, and a comparison of amastigotes with extracellular organisms (promstigotes), and biological activity in animal models and clinical settings.

## 5. Conclusions

In this report, IONPs were synthesized via a green method. IONPs were mainly spherical and homogeneous with an average size of about 4 nm and were synthesized via an eco-friendly, simple, and inexpensive method. The UV-visible spectrophotometer absorption peak was at 334 nm.

## Figures and Tables

**Table 1:** Observed bands in Fourier transform infrared analysis and related functional groups.

Band (cm^−1^)	Assigned or Associated	Reference
556 and 472	Metal oxygen (Fe–O)	[40]
1036	C–O	[10]
1277	C–N aromatic and aliphatic amines	[41]
1632	Carbonyl (–C=O) group stretching vibration	[42]
2362	CH stretching	[43]
2926	Asymmetric stretching of C–H	[44]
3426	N–H stretching vibration of group NH_2_ and O–H	[42,45]
